# Testing Phylogenetic Hypotheses of the Subgenera of the Freshwater Crayfish Genus *Cambarus* (Decapoda: Cambaridae)

**DOI:** 10.1371/journal.pone.0046105

**Published:** 2012-09-26

**Authors:** Jesse W. Breinholt, Megan L. Porter, Keith A. Crandall

**Affiliations:** 1 Department of Biology, Brigham Young University, Provo, Utah, United States of America; 2 Department of Biology, University of South Dakota, Vermillion, South Dakota, United States of America; 3 Computational Biology Institute, George Washington University, Ashburn, Virginia, United States of America; University of Poitiers, France

## Abstract

**Background:**

The genus *Cambarus* is one of three most species rich crayfish genera in the Northern Hemisphere. The genus has its center of diversity in the Southern Appalachians of the United States and has been divided into 12 subgenera. Using *Cambarus* we test the correspondence of subgeneric designations based on morphology used in traditional crayfish taxonomy to the underlying evolutionary history for these crayfish. We further test for significant correlation and explanatory power of geographic distance, taxonomic model, and a habitat model to estimated phylogenetic distance with multiple variable regression.

**Methodology/Principal Findings:**

We use three mitochondrial and one nuclear gene regions to estimate the phylogenetic relationships for species within the genus *Cambarus* and test evolutionary hypotheses of relationships and associated morphological and biogeographical hypotheses. Our resulting phylogeny indicates that the genus *Cambarus* is polyphyletic, however we fail to reject the monophyly of *Cambarus* with a topology test. The majority of the *Cambarus* subgenera are rejected as monophyletic, suggesting the morphological characters used to define those taxa are subject to convergent evolution. While we found incongruence between taxonomy and estimated phylogenetic relationships, a multiple model regression analysis indicates that taxonomy had more explanatory power of genetic relationships than either habitat or geographic distance.

**Conclusions:**

We find convergent evolution has impacted the morphological features used to delimit *Cambarus* subgenera. Studies of the crayfish genus *Orconectes* have shown gonopod morphology used to delimit subgenera is also affected by convergent evolution. This suggests that morphological diagnoses based on traditional crayfish taxonomy might be confounded by convergent evolution across the cambarids and has little utility in diagnosing relationships or defining natural groups. We further suggest that convergent morphological evolution appears to be a common occurrence in invertebrates suggesting the need for careful phylogenetically based interpretations of morphological evolution in invertebrate systematics.

## Introduction

The Cambaridae are comprised of 12 freshwater crayfish genera, of which three are species rich (greater than 90 species each), namely *Cambarus*, *Orconectes*, and *Procambarus* [1]. All three of these species rich genera have been divided into a variety of subgenera based on mainly morphometric considerations. Typically, no synapomorphic characters are offered to define subgenera leaving them suspect from phylogenetic and diagnostic perspectives. The subgenera designations in *Procambarus* and *Orconectes* are based on form one male gonopod morphology (the gonopods of crayfish males in breeding form) [2]. However, the subgenera in the genus *Cambarus* are mainly based on chelae morphology [3]. Crandall & Fitzpatrick [4] tested hypotheses of subgenera monophyly and species relationships within the genus *Orconectes* and found that subgeneric designations do not reflect phylogenetic relationships estimated with molecular sequence data. However, subgeneric relationships in the other two species rich genera, *Procambarus* and *Cambarus*, have not been tested to date. Our study will focus on the subgenera of *Cambarus* where phylogenetic hypotheses are based mainly on chelae morphology [3]. Thus, using the genus *Cambarus* we test the utility of morphological subgeneric designations based on traditional taxonomy within a robust phylogenetic framework.

**Figure 1:**
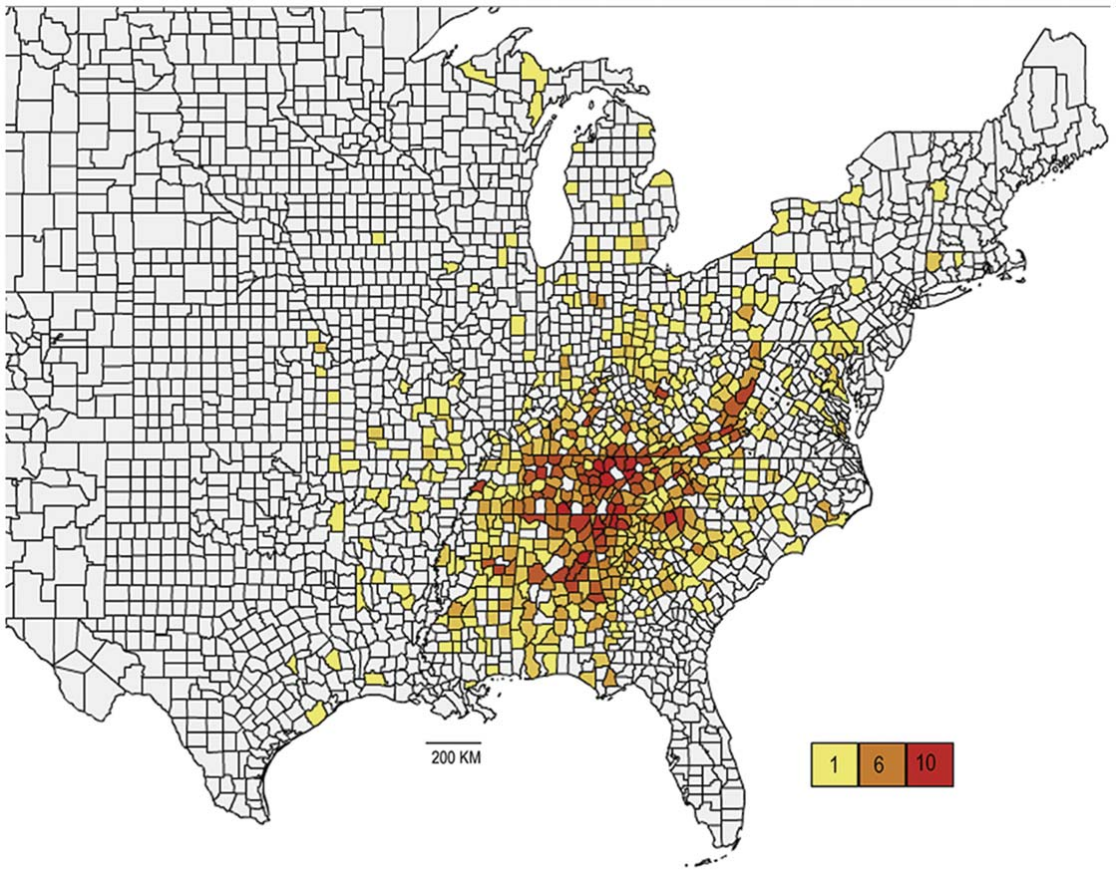
Choropleth map of the distribution of *Cambarus* created with the open source web tool Openheatmap (http://www.openheatmap.com/) using counts of species in each United States county collected from the SI USNM Invertebrate collection data base (downloaded February, 2012 from http://collections.mnh.si.edu/search/). Counties are colored according to the number of species in that county listed in the SI USNM records with a color scheme scale shown in the lower right corner of figure.

The genus *Cambarus* [5], family Cambaridae, infraorder Astacoidea, consists of 12 subgenera (*Aviticambarus*, *Cambarus*, *Depressicambarus*, *Erebicambarus*, *Exilicambarus*, *Glareocola*, *Hiaticambarus*, *Jugicambarus*, *Lacunicambarus*, *Puncticambarus*, *Tubericambarus*, and *Veticambarus*) [3], [6], [7], [8] and approximately 104 species [9], [10]. *Cambarus* ranges from the coastal region of New Brunswick, Canada, south to the Florida panhandle, west to Texas, and northward to Minnesota and southern Ontario, Canada [11]. The center of diversity for the genus is in the Southern Appalachians of the United States (Fig. 1) [3]. Approximately half of the Cambarus species are either listed as endangered, threatened, or vulnerable [12], [13]. Therefore, resolving taxonomy is an important step in the management and conservation of these endangered crayfish species [14]. Furthermore, the establishment of a robust phylogeny for taxa with well articulated conservation status can greatly aid in defining and prioritizing areas for conservation (e.g., [15]).

The *Cambarus* species inhabit three general habitat types: streams, burrows, and caves [3]. While a few subgenera are restricted to species that have the same habitat type (e.g., caves: *Aviticambarus*, streams: *Exilicambarus*, *Glareocola*, *Hiaticambarus*, *Veticambarus*, and burrows: *Lacunicambarus*, *Tubericambarus*), the rest of the subgenera are made up of species with a mix of habitats (e.g., *Jugicambarus* species inhabit streams, burrows, and caves). In his treatment of this group in 1969, Hobbs [3] proposed a phylogenetic hypothesis for the *Cambarus* subgenera based on chelae morphology. While Hobbs [3] also used carapace features he clearly indicates his reliance on chelae morphology when he stated, “With such marked parallel evolution occurring in the several basic stock of the genus, were it not for the chelae and certain more subtle features of the structures just mentioned, it is doubtful that any sort of evolutionary lineages could have been conceived [3].” Remarkable for the time (1969), Hobbs' [3] *Cambarus* hypothesis of evolutionary relationships among the subgenera is depicted by a tree and is illustrated with the chelae of the nominal species for each subgenus.

**Figure 2:**
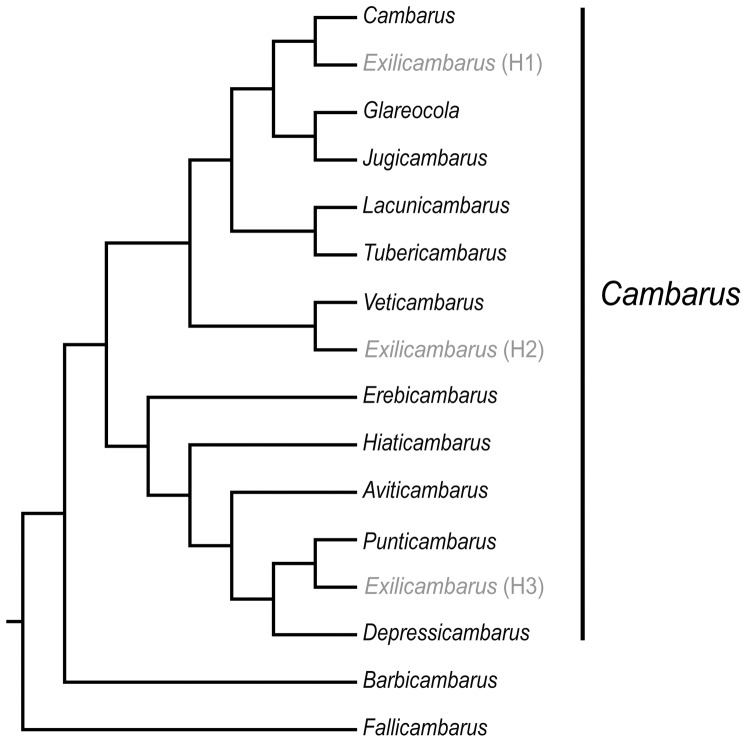
Hypothesized relationships among subgenera of the genus *Cambarus* based on Hobbs [**3**] with modification to include subgenera described and/or modified post Hobbs [**3**]. *Tubericambarus* and *Glareocola* were placed as sister to the subgenera from which they were partitioned [6], [7]. The monotypic subgenus *Exilicambarus* is placed in three locations (three possible topologies H1, H2, and H3) based on Bouchard and Hobbs' [8] assessment of potential sister taxa.

Since Hobbs [3], the subgenus *Barbicambarus* [16] was raised to genus status in 1972, a new monotypic subgenus, *Exilicambarus*, was designated in 1976 [8], and two subgenera were further partitioned from existing subgenera, namely *Glareocola* (from *Jugicambarus*) in 1995 [7] and *Tubericambarus* (from *Lacunicambarus*) in 1993 [6], [7]. Due to the unique suite of characters of *Exilicambarus*, its relationship to other genera was difficult to assess; so Bouchard and Hobbs [8] hypothesized *Exilicambarus* may be closely related to one of the following three subgenera: *Jugicambarus*, *Puncticambarus*, or *Veticambarus*. Neither Jezerinac [6] nor Bouchard and Bouchard [7] discussed specific phylogenetic relationships of their new subgenera *Tubericambarus* and *Glareocola*, but we presume they would be sister to those subgenera within which they were contained before the partitioning into new subgenera. Therefore, starting with Hobbs' [3] phylogenetic hypothesis with adjustments to account for the new subgenera within the genus, our current hypothesis for the phylogenetic relationships among the subgenera within the genus *Cambarus* based on morphology is represented by three possible topologies (Fig. 2: H1, H2, and H3) that differ only in the placement of *Exilicambarus*.

Our study tests this updated phylogenetic hypothesis using a multigene data set with likelihood and Bayesian optimality criteria. We sample all the subgenera and approximately 68% of the described species. To test the utility of subgeneric designations based on traditional taxonomy, we use our resulting phylogeny to test the monophyly of the subgenera as well as the phylogenetic relationships among the subgenera (Fig. 2). In order to better understand the evolutionary history of *Cambarus*, we use a multiple model regression analysis to test three possible explanatory variables (taxonomic model, habitat model, and geographic model) for significant correlation with estimated phylogenetic relationships. We discuss the implications of our results on the interpretation of morphological evolution within Cambaridae and on current taxonomy. Lastly, we discuss convergent evolution in invertebrates and the implications for taxonomic and systematic studies.

## Methods

### Taxon sampling, DNA extraction, PCR, and sequencing

Crayfish species were sampled to represent all 12 of the *Cambarus* subgenera for a total of 71 out of the 104 described species. We obtained complete taxon sampling for five subgenera and sampled at least 52% for the other subgenera, excluding *Tubericambarus* from which only one of the three species was sampled (Table 1 and Appendix S1). For two species, *Cambarus* (*L.*) *diogenes* and *Cambarus (D.) striatus*, we include several localities across their geographic range to examine the diversity and phylogenetic placement of samples because these are broadly distributed species suspected of being species complexes [2]. Outgroup taxa were chosen to provide a broad representation of the genus diversity in Astacoidea allowing us to test the monophyly of the genus *Cambarus*, and included species from the following genera: *Astacus*, *Pacifastacus*, *Cambarellus*, *Procambarus*, *Orconectes*, *Faxonella*, *Fallicambarus*, and *Barbicambarus* (Appendix S1).

**Table 1:** The number of *Cambarus* species sampled by subgenus.

Subgenus	Described species	Species sampled
*Aviticambarus*	6	5
*Cambarus*	10	5
*Depressicambarus*	17	12
*Erebicambarus*	5	5
*Exilicambarus*	1	1
*Glareocola*	3	3
*Hiaticambarus*	9	8
*Jugicambarus*	24	16
*Lacunicambarus*	3	3
*Puncticambarus*	*21	11
*Tubericambarus*	*4	1
*Veticambarus*	1	1
Total	104	71

* Includes recently described *Cambarus* species by Cooper and Price [77], Loughman et al. [78], and Thoma [79].

Crayfish collection, tissue preservation, and DNA extraction followed protocols and methods described in Porter et al. [17] and Crandall & Fitzpatrick [18]. A few species are represented by tissue taken from vouchered specimens from the Smithsonian National Museum of Natural History (USNM prefix) or the North Carolina State Museum of Natural History (NCSM prefix) (Appendix S1). Crayfish are invertebrates and no specific permits were required at the time of collection and localities were not privately owned and special permission is not required to access these locations. Tissues for cave crayfish were taken from the crustacean collection at the Monte L. Bean Life Science Museum at Brigham Young University where permit and information concerning access and property ownership are archived. The federally endangered cave crayfish *Cambarus* (*J.*) *aculabrum* was collected in 1992 before it was federally listed in 1993 and no permit was required for collection. Currently, The Nature Conservancy owns the cave where *Cambarus* (*J.*) *aculabrum* was collected; however, at the time of collection we received permission to access the cave from the private land owner.

Polymerase chain reaction (PCR) products for three mitochondrial genes - partial 16S rDNA (∼460 bp; using the primer 16sf-cray [19] and 16s-1472r [18]), partial COI (∼659 bp; with primers LCO1-1490 and HCO1-2198 [20]), and partial 12S rDNA (∼390 bp; using the primers 12sf and 12sr [21]) - were amplified using protocols following Porter et al. [17] and Crandall & Fitzpatrick [18]. We also PCR amplified the partial nuclear gene 28S rDNA (∼800–1000 bp; with primers 28s-rd3a and 28s-rD5b [22], [23] or with primers made for this study 28sF-cray 5′- TCGTCGGCTGTCGGCTGGGT -3′ and 28sR-cray 5′- CTAGATGGTTCGATTAGTCTTTC -3′ using an annealing temp of 65°C). Bidirectional sequences for each gene were generated on an ABI Prism 3730XL capillary sequencer using the ABI Big Dye Ready-Reaction kit following standard cycle sequencing protocols, with an exception of 1/16th of the standard reaction volume. The mitochondrial genes 16S, COI, and 12S have differing amounts of variation and are commonly used for phylogenetic analysis in crayfish [19], [24], [25], [26], [27]. The region of 28S used tends to be the most variable region of 28S among Crustacea [24] and was sampled for a subset of taxa to serve as a nuclear genome marker for estimating phylogenetic relationships among the species of the genus *Cambarus*. Sequence data for the outgroups and several *Cambarus* taxa were obtained from GenBank (49 sequences), and the remaining sequences (263 sequences) were generated in the Crandall lab as described above with GenBank accession numbers provided in Appendix S1. Due to the common problem of amplifying nuclear mitochondrial pseudogenes (numts) when amplifying COI, we followed Song et al. [28] and Buhay [29] by checking PCR results with gel electrophoresis post PCR, translating sequences to check for indels and stop codons, and comparing sequences to closely-related species.

### Sequence analyses

Sequencher 4.9 (GeneCodes, Ann Arbor, MI, USA) was used to assemble and clean the sequences bidirectionally, as well as check for stop codons in the COI gene. Each gene was aligned separately using MAFFT [30], [31] with the G-INS-I alignment algorithm for the full data set (*Cambarus* taxa and outgroup taxa) and for only *Cambarus* taxa. MAFFT was used because the iterative algorithm allows for fast and repeatable alignments. The best fit model of evolution was estimated with MODELTEST 3.7 [32] for each gene alignment using the Bayesian information criterion (BIC) [33]. Pairwise model corrected genetic distances were calculated for each gene for all of our samples of *Cambarus* taxa in PAUP* v. 4.02b [34], for which we report the mean genetic distance in order to compare the relative amounts of divergence of each gene.

### Phylogenetic analyses and hypothesis testing

Maximum likelihood (ML) and Bayesian optimality criteria were used to estimate phylogenies using RAxML [35] and MrBayes [36]. Both RAxML and MrBayes allow for data partitioning, increasing the accuracy and ability to account for gene specific rates and nucleotide heterogeneity; therefore, independent models were given to each gene in the concatenated analyses. For RAxML we used the GTR+G model over the only alternative, GTR+I+G, following the author's suggestions that the GTR+I+G may cause problems in model parameter optimization. Each gene was analyzed independently in RAxML using a combined ML topology search and bootstrap pseudoreplication estimation for 1000 bootstraps to determine nodal support [37]. Gene trees were compared to identify taxa with highly supported conflicting placement among mitochondrial genes, and sequences that fit this criterion were not included in further analysis or in Appendix S1. For the concatenated ML analysis, we executed 200 tree searches starting from random tree topologies, as well as ML searches using every fifth bootstrap pseudoreplication out of 1000 as a starting topology. The tree with the best ML score was selected and we assessed confidence in nodal support through 1000 bootstrap pseudoreplications estimated in RAxML. Our Bayesian analyses were performed using MrBayes with two independent runs with one cold chain and seven hot chains. Each run was started from a random tree using the default flat priors for 1×10^7^ generations sampling every 1000 generations. We unlinked the variables statefreq, revmat, shape, and pinvar for all gene models and the numbers of parameters (nst) and rate heterogeneity (G, I or G+I) were assigned to each gene following the ModelTest BIC results. Split frequencies below 0.01 as well as examining the negative log likelihood posterior distribution between runs were used to check for convergence and determine the length needed for burnin using MrBayes output and the program Tracer v1.5 [38]. The two MrBayes runs were combined after the deletion of burn-in and a majority rule consensus tree was created with nodal confidence for the trees assessed using node posterior probabilities. We made choropleth maps to represent the geographic distribution of species in resulting clades from the Bayesian analysis created with the open source web tool Openheatmap (http://www.openheatmap.com/) using counts of species with distributions in each state. Geographic distributions for each species were taken from Fetzner [10] and Hobbs [2] and are listed in Appendix S2.

To test phylogenetic hypotheses of subgeneric monophyly and relationships, we compared the best resulting ML topology to topologies constrained to fit alternative hypotheses using the approximately unbiased test (AU) [39] in the program CONSEL [40]. Constraint topologies were estimated in RAxML with the −g constraint option using the best scoring tree for topology tests estimated from 200 ML searches starting from random tree topologies. In addition to ML topology tests, Bayesian topological tests (*P*p) were performed following Huelsenbeck et al. [41].

### Taxonomic, geographic, and habitat correlation with phylogenetic distance

We used an adaptation of Manly [42] code for multiple regression that accounts for pairwise symmetrical matrices in the program FSTAT [43] to test for significant correlation and explanatory power of geographic distance, taxonomic model, and a habitat model to estimated phylogenetic distance. The phylogenetic distance matrix was estimated from our Bayesian topology in Mesquite [44] using pairwise node distance for all *Cambarus* taxa included in this study. We used pairwise node distance over nucleotide distance, as taxa with missing genes would influence genetic distance estimates. The taxonomic distance model matrix was built using current taxonomy with a distance of zero assigned to pairs of taxa within the same subgenus and a distance of one to pairs of taxa from different subgenera. The habitat distance matrix consisted of pairwise comparisons of *Cambarus* taxa with a distance of zero for taxa that share the same habitat type and a distance of one for pairs that differ. Habitat information for each species was taken from Fetzner [10] and Hobbs [2] and is listed in Appendix S2. Species that occupy multiple habitats such as *Cambarus tenebrosus* (streams and caves – see [45]) were considered equally close to stream and cave taxa and given a distance of zero for comparisons of taxa with these habitat types. Geographic distance between two *Cambarus* samples was estimated in meters from longitude and latitude in the program DIVA-GIS 7.4 (www.diva-gis.org).

## Results

**Figure 3:**
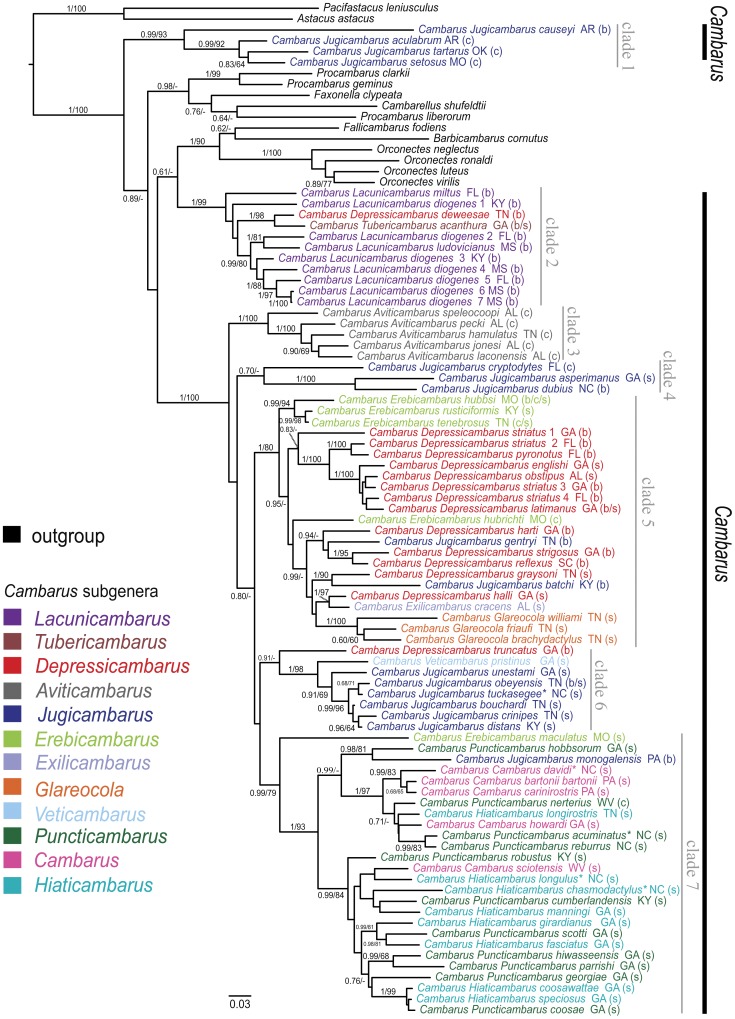
Bayesian estimate of phylogenetic relationships amongst the species and subgenera of the crayfish genus *Cambarus* with outgroups from other genera within the family Cambaridae. Taxa labels are followed by the US state the sample was collected in, the type of habitat of each species in parenthesis (c = cave, b = burrow, s = stream), and in some cases a number for species with more than one sampled individual. Nodal support is indicated by Bayesian posterior probabilities before the slash and ML bootstrap values after the slash on branches leading to the supported node.

**Figure 4:**
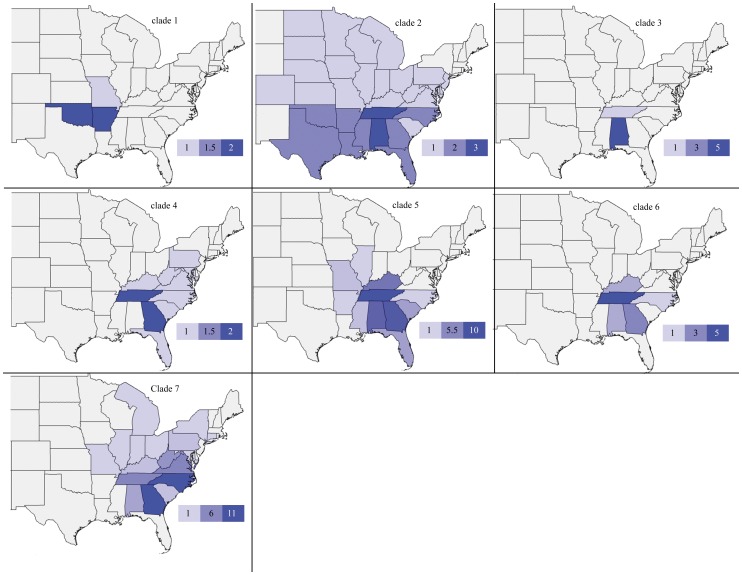
Choropleth maps to represent the geographic distribution of species in resulting clades from the Bayesian analysis (**Fig. 3**) created with the open source web tool Openheatmap ( http://www.openheatmap.com/ **) using counts of species with distributions in each state.** States are colored according to the number of species with distributions in that State with a color scheme scale shown in the lower right corner of each map.

Examination of COI sequences for stop codons yielded no identifiable pseudogenes (however, see below). The best fit nucleotide model of evolution for the *Cambarus* species data sets were HKY+I+G for COI, 16S, and 12S and F81 for 28S. The mean model-corrected sequence distance for genes within the genus *Cambarus* indicate that COI (18.7%) is the most variable followed by 16S (14.4%) and then 12S (11.6%) with very little divergence in 28S (0.3%). While 28S did provide some resolution for the group, compared to even the slowest mitochondrial gene the divergence in 28S is minimal and had little utility for use within this group. For the entire data set (*Cambarus* and outgroups) BIC indicated that the best model was a two-parameter model for COI with rates = invgamma and a six parameter model for the other three genes with rate = invgamma for 16S and rates = propinv for 12S and 28S. Examination of gene trees resulted in the identification of six taxa with mitochondrial gene sequences having strongly supported phylogenetic placement in conflict. These sequences included two sequences for each mitochondrial gene. One was likely contamination as it was exactly the same as another taxon in the data set and the five others may have been pseudogenes [see citation 29 for a detailed study on pseudogenes in crayfish] and were removed from the data set. Gene trees resulted in no strongly supported nodes in conflict between the mitochondrial and nuclear gene justifying concatenation of the data set. The two independent MrBayes runs converged and we set burnin at 4×10^6^ where the negative log likelihood posterior distribution converged and split frequencies were below 0.01 for a total of 12,000 trees in our posterior post-burin distribution. The concatenated ML and Bayesian analysis resulted in trees with similar topologies. We chose to present our Bayesian topology (Fig. 3) as it represents a distribution of likely topologies instead of the single topology (for comparison we include our ML topology in Appendix S3). The major clades in the ML and Bayesian analysis contained the same taxa, but relationships among those taxa with little to no support changed between the two methods. The largest difference between the ML and Bayesian results was the poorly supported sister grouping of *Lacunicambarus* taxa to a clade of outgroup taxa containing *Barbicambarus cornutus* in the Bayesian topology, which is not found in the ML results. Both optimality criteria resulted in a paraphyletic *Cambarus*. Subgenera with multiple species represented, excluding *Glareocola* and *Aviticambarus*, also resulted in paraphyletic or polyphyletic assemblages. The geographic distributions of clades 2–6 (Fig. 4) have their highest species density in or near the Cumberland plateau region with considerable overlap in the distribution of individual species. The AU and p*P* topology tests reject (Table 2) all three proposed phylogenetic hypotheses based on Hobbs [3] and subsequent work (H1–H3 see Fig. 2). The monophyly of genus *Cambarus* is not rejected by the AU test, yet in the Bayesian topology test a monophyletic *Cambarus* is found in only 0.2% of the post-burnin tree distribution. Subgeneric AU and p*P* topology tests result in the rejection of monophyly of six subgenera (*Cambarus*, *Depressicambarus*, *Erebicambarus*, *Hiaticambarus*, *Jugicambarus*, *Puncticambarus*) (Table 2). For *Lacunicambarus* the AU test failed to reject monophyly (Table 2); however, a monophyletic *Lacunicambarus* was represented in only 4.03% of the post-burnin posterior probability distribution. Even with limited sampling throughout their range, *Cambarus diogenes* and *Cambarus striatus* were not monophyletic and likely represent more than a single species.

**Table 2:** Results from approximately unbiased test (AU) test and Bayesian topological tests (*P*p) topology hypothesis tests.

Hypothesis	AU p-value	*P*p	N # of trees
H1	0.0000	0.00%	0
H2	0.0000	0.00%	0
H3	0.0000	0.00%	0
Genus *Cambarus*	0.2130	0.28%	33
Subgenera			
*Cambarus*	0.0000	0.00%	0
*Depressicambarus*	0.0000	0.00%	0
*Erebicambarus*	0.0280	0.00%	0
*Hiaticambarus*	0.0000	0.00%	0
*Jugicambarus*	0.0000	0.00%	0
*Lacunicambarus*	0.4710	4.03%	484
*Puncticambarus*	0.0000	0.00%	0

Table includes the p-value for the AU test and the number (N # of trees) and percent (*P*p) of trees that fit the given hypothesis from the post-burnin set of Bayesian trees.

The multiple regression model resulted in two variables (taxonomic model and geographic distance) with significant correlation to phylogenetic distance (Table 3). However, the taxonomic model had the most explanatory power. Habitat was not significantly correlated with phylogenetic distance (Table 3).

**Table 3:** Results from multiple regression model for correlation of three variables (Taxonomic model, Geographic distance, Habitat model) to Phylogenetic distance (node distance from Bayesian topology).

The total sum of square for phylogenetic distance: 52643.5469					
Variable	Partial Corr.	Beta	SS	P(Beta)	P(SS)
Taxonomic model	0.424695	5.518281	9495.1016	0.0001	0.0001
Geographic distance	−0.148457	−0.002056	1160.2305	0.0001	0.0001
Habitat model	0.004542	0.040158	1.0859	0.756	0.7964
Error sum of squares: 41987.1289					
Percent of the variance explained by the model: 20.24					

Partial Correlation (Partial Corr.), Coefficient (Beta) and Sum of squares (SS) for the observed data. P-values after 10000 randomizations for absolute regression coefficients (2-sided) P(Beta) and for extra sums of squares P(SS).

## Discussion

### The genus *Cambarus*

Our resulting phylogenetic estimate among species from the crayfish genus *Cambarus* found incongruence between taxonomy and the estimated evolutionary history, with six subgenera out of ten being rejected as monophyletic groups. We also rejected all variations (H1–H3) of our updated phylogenetic hypothesis for the relationships among the subgenera in this group. Our resulting phylogenetic estimate provides strong support for the previous designation of *Barbicambarus* as a distinct genus from *Cambarus* in the early 1970's [16]. The subgenera of *Cambarus* are predominantly nonmonophyletic suggesting a lack of utility for current subgeneric designations as diagnosing species relatedness or defining natural groups. Our phylogenetic estimate of relationships among the subgenera of *Cambarus* showed that a single clade of *Jugicambarus* species (clade 1, Fig. 3 and Fig. 4) that is geographically disjunct from the main distribution of the genus (they occur in Missouri and Arkansas contrasted with the main distribution of *Cambarus* in the Southern Appalachians) causes the genus to be nonmonophyletic. However, the statistical support for this conclusion is weak and we fail to reject the null hypothesis of *Cambarus* monophyly. Excluding these geographically disjunct *Jugicambarus* species, our phylogeny is not inconsistent with Hobbs' [3] hypothesis that the geographic origin of the genus lies near eastern Tennessee in the Cumberland plateau with each well-supported clade having species with distributions in this region (Fig. 4). Another clade containing all the representatives of *Lacunicambarus* (clade 2, Fig. 3 and Fig. 4) is also very weakly paraphyletic with respect to the major group of *Cambarus* species. We fail to reject the monophly of *Lacunicambarus* with an AU test despite *C.* (*T.*) *acanthura* and *C.* (*D.*) *deweesae* placement within the *Lacunicambarus* clade. *Cambarus* (*L.*) *diogenes* representatives in the *Lacunicambarus* clade are polyphyletic; however, this species is known to represent a species complex [2] and has the largest geographic distribution of the *Cambarus* species. The distributions of species in clades 3–7 overlap considerably (Fig. 4). Although the states with the most species are likely to change when including unsampled *Cambarus* taxa, it is clear from the species sampled that species diversity decreases as a function of distance from the Cumberland plateau region (Fig. 4). While we fail to reject the monophyly of *Cambarus*, Buhay and Crandall [19] show several of the *Orconectes* cave species are closely related to *Cambarus* taxa and including these cave species may lead to the rejection of monophyly of *Cambarus*.

### The subgenera within *Cambarus*

Two subgenera form monophyletic groups, *Aviticambarus* (clade 3, Fig. 3) and *Glareocola* (nested within clade 5, Fig. 3), and are highly supported, and interestingly, habitat type is conserved within these subgenera. The cave species within *Aviticambarus* are basal to the robust clade of the majority of the species of *Cambarus*. This is consistent with previous arguments on the basal position of *C.* (*A.*) *pecki* (reviewed in [46]). The species within the monophyletic *Glareocola* are found in the gravel substrate of fast flowing streams of the Highland Rim. Their highly supported monophyly and phylogenetic distinctiveness supports their designation as a subgenus (Bouchard and Bouchard, 1995). The subgenus *Tubericambarus* (for which we only have a single species represented in our study – *Cambarus* (*T.*) *acanthura*) was nested within *Lacunicambarus* from which the subgenus was partitioned, suggesting that the subgenus designation may be unwarranted.

The two monotypic subgenera *Exilicambarus* and *Veticambarus* are nested in clades comprised of taxa from multiple subgenera (clade 5 and 6, respectively, Fig. 2). Hobbs [3] hypothesized that the subgenus *Veticambarus* may be the ancestor to all the members of the genus, due to the many primitive morphological features and location in the Cumberland Plateau region (hypothesized as the origin of the genus). Hobbs [3] considered *Veticambarus* chela features ancestral and used this to polarize his proposed sequence of chela evolution for *Cambarus*. Had Hobbs been correct, we would expect *Veticambarus* to be the oldest lineage and basal to the other species in the genus. However, we find *Veticambarus* to be a fairly derived member of the genus, forming a highly supported clade including species from the subgenus *Jugicambarus* living in similar habitats (streams) to *Veticambarus*.

The subgenus *Jugicambarus* is rejected as being monophyletic with representatives falling out in five clades (clades 1, 4, 5–7, Fig. 3) throughout the tree, with well-supported sister relationships with three subgenera (*Puncticambarus*, *Veticambarus*, and *Depressicambarus*). Excluding cave species and *Cambarus* (*J.*) *carolinus* (not sampled), *Jugicambarus* species are united morphologically by the mesial surface of the palm having a single row of cristiform tubercles, the dorsal surface of chela being deeply pitted, and both palms and fingers frequently bearing stiff setae, with well defined latitudinal ridges dorsally on the fixed finger of the chela. These chela features used by Hobbs [3] to unite the *Jugicambarus* appear to be convergent, as this combination of features are contained by *Jugicambarus* species in several different clades in the phylogeny. None of the burrowing *Jugicambarus* species sampled group together in the phylogeny. The three *Jugicambarus* burrowers that do not group with other *Jugicambarus* species, *C.* (*J.*) *gentryi*, *C.* (*J.*) *batchi*, and *C.* (*J.*) *monogalensis*, have strongly supported sister relationships with burrowing species from other subgenera (*Puncticambarus* and *Depressicambarus*).

*Erebicambarus* (all species in clade 7, Fig. 3) is also rejected as monophyletic and is one of the subgenera for which we obtained complete sampling of the described species. The strictly troglobitic crayfish species C. (*E.*) hubrichti and the stream species C. (*E.*) maculatus are polyphyletic with respect to the main cluster of species in this group. Hobbs and Pflieger [47] hypothesized that the highly similar morphology of C. (*E.*) maculatus, C. (*E.*) rustiformicus, and C. (*E.*) hubbsi was due to the maintenance of shared ancestral states through the continual use of similar habitat (parallel evolution) and not independently evolved due to similar habitat (convergent evolution). However, we find strong evidence for the latter, with strong branch support for the two different clades that contain these species. Cambarus (*E.*) maculatus, C. (*E.*) rustiformicus, and C. (*E.*) hubbsi are very similar morphologically and the only diagnosable difference in form two males and females of these species is the speckled coloration of C. (*E.*) maculatus [47]. This is yet another illustration of how much influence convergent evolution has had on the evolution of the morphology in this group. Depressicambarus (species found in clades 2, 5–6, Fig. 3) is also rejected as monophyletic, with taxa generally falling out in two clades in the same portion of the tree, excluding the two disjunct members C. (D.) deweesae and C. (D.) truncatus. The inclusion of several C. (D.) striatus samples from the southern distribution of this species confirms the suspicions that this species represents a species complex [2], [3], [48].

### Morphological implications

While chela morphology were key characters in Hobbs' [3] hypothesis of *Cambarus* subgenus relationships, he also relied on several other morphological features such as type and distribution of punctuations on the areola of the carapace. It was the combination of such features in each subgenus that led Hobbs [3] to hypothesize that the subgenera were natural groups. Hobbs [3] specifically pointed out that as morphologically diverse as the *Puncticambarus* species may seem, the combination of morphological traits provide overwhelming evidence that it represents a natural group. We reject the monophyly of *Puncticambarus* with an AU test, with members in two clades (within clade 7, Fig. 3) highly supported as sister groups containing all the sampled species from the subgenera *Cambarus*, *Hiaticambarus*, and *Puncticambarus*. The species in these three subgenera are all found in lotic habitats (excluding the troglobitic *C.* (*P.*) *nerterius* and *C.* (*C.*) *ortmanni*, a secondary burrower not sampled in our study) and appear to be nearly equally distributed between two clades (within clade 7, Fig. 3) in our phylogeny. Given our phylogeny, the following combinations of chela and carapace features used by Hobbs [3] to define these three subgenera clearly demonstrate that these features do not define natural groups: 1. Number of rows of tubercles on the mesial surface of the palm with one row in *Hiaticambarus*, two rows in *Puncticambarus*, and one to two rows in *Cambarus*, with the second row strongly depressed if present; 2. Presence of well-defined latitudinal ridges dorsally on the fixed finger of the chela in *Puncticambarus* and *Cambarus* and not in *Hiaticambarus*; 3. Conspicuous tufts of setae present at the mesial base of the fixed finger in *Hiaticambarus*, but absent in *Cambarus* and *Puncticambarus*; 4. Carapace areola punctuations sparsely to moderately punctate in *Cambarus*, densely studded with shallow punctuations in *Puncticambarus*, and crowded with deep punctuations in *Hiaticambarus*. The phylogenetic results of our study clearly show that neither single nor combinations of morphological traits used to delimit *Cambarus* subgenera define natural groups or can be used to evaluate evolutionary relationships within the defined subgenera.

### Taxonomic, geographic, and habitat correlation with phylogenetic distance

In order to better understand the evolutionary history of *Cambarus*, we applied a multiple model regression analysis to test three possible explanatory variables for significant correlation with estimated phylogenetic relationships. Ironically, this multiple regression model indicates taxonomy has the most explanatory power of genetic relationships. While the monophyly of the subgenera can be strongly rejected, small groups of members of the subgenera are monophyletic. Therefore, taxonomy is somewhat useful in predicting relationships and should be used to direct sampling schemes for studies within this group. While geography is a significant variable, it has a negative value and represents a very small part of the explanatory power of the model. The negative correlation indicates that geographic distance decreases with node distance. The correlation of geographic distance and genetic relationships is likely affected by incomplete sampling, especially the large geographic distance ∼400 km between sister species (node distance of 1). However, this analysis clearly shows geography cannot be used to predict relationships, as the standard isolation by distance model (Wright 1943) does not fit the history of speciation in this group. Surprisingly, habitat was not a significant contributor to the model, showing that differing or similar habitats cannot be used to predict phylogenetic relationships within *Cambarus*, despite a variety of small clusters of species by habitat type. An examination of the distribution of habitat type across our phylogenetic tree (Fig. 2) shows that each habitat type occurs in several clades throughout the tree. Even with our incomplete sampling of the genus, it appears that habitat has played an important role in the evolution of this group with each habitat type appearing several times in the phylogeny. The role of habitat in the evolution of this group should be studied further with complete sampling of *Cambarus* taxa to allow for a robust ancestral reconstruction of habitat history.

### Conclusions for cambarid crayfish

Our results suggest that convergent evolution has impacted the morphological features used to delimit the *Cambarus* subgenera, as relationships based on chelae morphology and carapace morphology are incongruent with estimated phylogenetic relationships. Much of the systematics within Cambaridae is based on form one male gonopod morphology. This is particularly true for the other two species rich genera *Orconectes* and *Procambarus*. Several molecular phylogenetic studies [4], [25], [49], [50], [51], [52] have suggested that gonopod morphology is the result of convergent evolution with respect to molecular phylogenetic estimates. Thus, while the lack of monophyly is consistent with the results of Crandall & Fitzpatrick [4] for the subgenera of *Orconectes*, the convergent morphologies used to diagnose those subgenera (gonopod morphology for *Orconectes* versus chelae and carapace morphology for *Cambarus*) are different. This suggests that subgeneric morphological diagnoses based on traditional cambarid crayfish taxonomy (form one male gonopods and combination of chela and carapace characters) might be confounded by convergent evolution across the cambarids. Thus, a wider suite of morphological characters under less selection from the environment than those used by traditional crayfish taxonomy should be assessed in future evaluations of evolutionary relationships among cambarid species. The use of molecular based phylogenies may be useful in evaluating synapomorphic morphological characters that reflect evolutionary relationships and are less affected by convergent evolution.

While one goal of systematic studies is to revise taxonomy to reflect evolutionary history, for *Cambarus* this task seems unwise without complete taxon sampling. In this group of crayfish, placement of unsampled species in any one lineage cannot be done with any degree of confidence. For example, the eight unsampled taxa from *Jugicambarus* could fall out in a new clade or in any one of five clades estimated with *Jugicambarus* species in them. This problem extends to all other subgenera without complete sampling (i.e., *Depressicambarus*, *Hiaticambarus*, *Puncticambarus*, and *Tubericambarus*) as well.

Future work in this genus specifically needs to obtain complete taxon sampling as well as increased sampling throughout the geographic range of each species. Other studies that have conducted extensive sampling within species from the genera *Orconectes*, *Procambarus* and *Cambarus* have also found significant population structure and cryptic diversity [45], [46], [50], [53], [54]. This suggests that extensive sampling within species is critically important for all cambarid crayfish before we can make meaningful (reflecting evolutionary history) and lasting taxonomic changes.

### Morphological convergence in invertebrates

Moore and Willmer's [55] review of morphological convergence suggested that convergent evolution was more common in invertebrates than previously thought. Since this review convergent evolution has been continually documented in taxa representing all major invertebrate lineages (e.g., [4], [50], [55], [56], [57], [58], [59], [60], [61], [62], [63], [64], [65], [66], [67], [68]). Convergent morphology resulting in taxonomy that does not reflect evolutionary history is not unique to crayfish and is commonly found in many invertebrate groups (e.g., [4], [68], [69], [70], [71], [72], [73], [74], [75], [76]). The central question of Moore and Willmer's [55] review was “How common is convergence in invertebrates?” Our results coupled with others across invertebrate diversity suggest convergent evolution is much more common than previously thought. We therefore recommend that the standard assumption of ‘no convergent evolution’ in morphological features defining taxonomic groups be rigorously tested in a robust phylogenetic framework when performing systematic studies.

## Supporting Information

Appendix S1 Table of collection data consisting of species name, collection number, locality data, and GenBank accessions for each gene.
(XLS)

Appendix S2 Table including the distribution of each species by US state and habitat of each species taken from Fetzner [10] and Hobbs [2].
(XLS)

Appendix S3 Figure of maximum likelihood estimate of phylogenetic relationships amongst the species and subgenera of the crayfish genus *Cambarus* with outgroups from other genera within the family Cambaridae. Taxa labels are followed by the US state the sample was collected in and in some cases a number for species with more than a single sample. Nodal support is indicated by Bayesian posterior probabilities before the slash and ML bootstrap values after the slash on branches leading to the supported node.
(TIF)
