# Supplementary figures and images for: Testing Phylogenetic Hypotheses of the Subgenera of the Freshwater Crayfish Genus Cambarus (Decapoda: Cambaridae)
Source: PLoS One. 2012 Sep 26;7(9):e46105. doi: 10.1371/journal.pone.0046105 (PMC3458831; doi:10.1371/journal.pone.0046105)

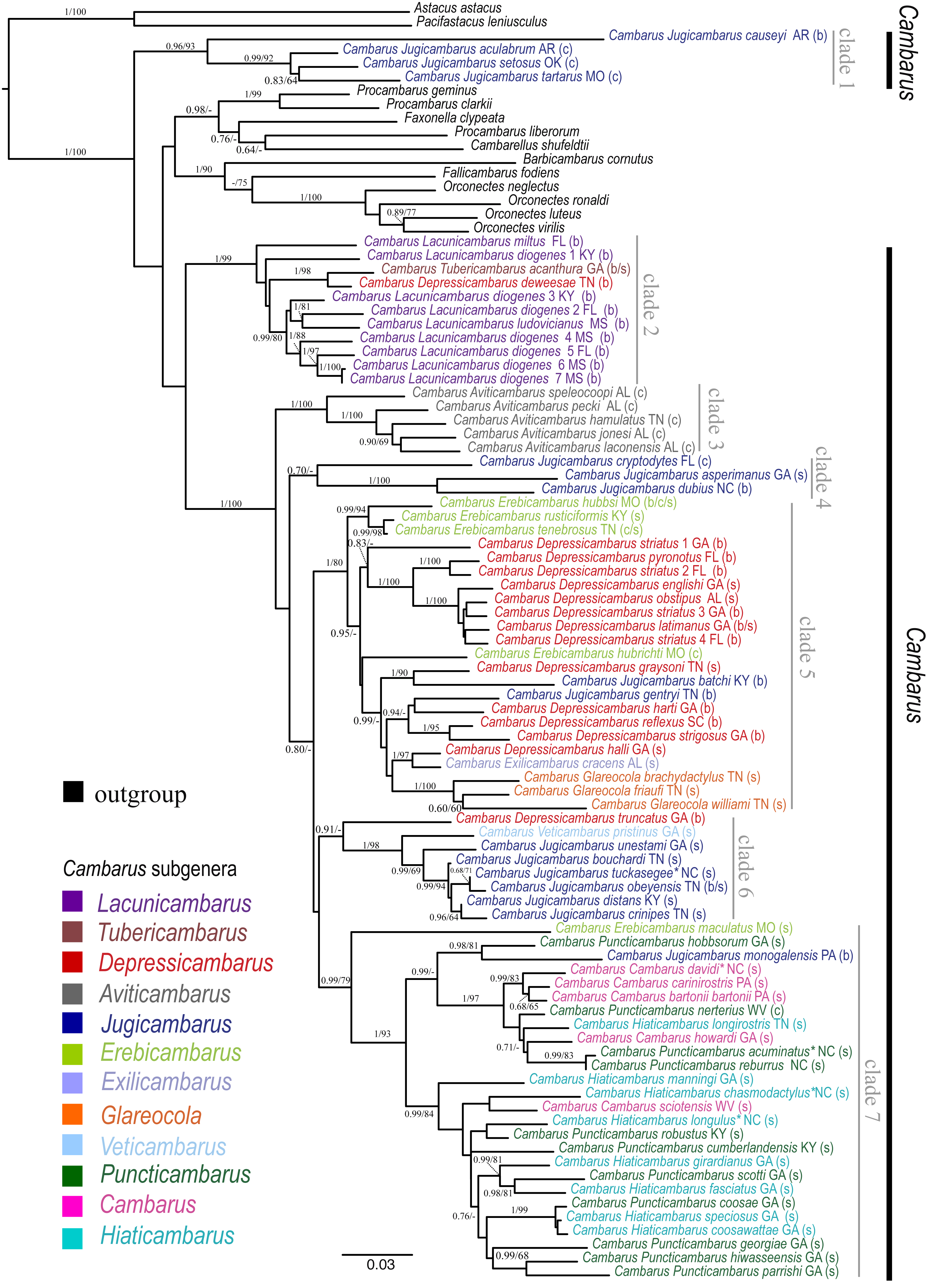

Supplement: Appendix S3 — Figure of maximum likelihood estimate of phylogenetic relationships amongst the species and subgenera of the crayfish genus Cambarus with outgroups from other genera within the family Cambaridae. Taxa labels are followed by the US state the sample was collected in and in some cases a number for species with more than a single sample. Nodal support is indicated by Bayesian posterior probabilities before the slash and ML bootstrap values after the slash on branches leading to the supported node. (TIF) [file pone.0046105.s003.tif]
